# COVID-19 apps in Singapore and Australia: reimagining healthy nations with digital technology

**DOI:** 10.1177/1329878X20949770

**Published:** 2020-11

**Authors:** Gerard Goggin

**Affiliations:** Nanyang Technological University, Singapore

**Keywords:** Australia, contact tracing apps, COVID-19, digital media, health communication, infectious diseases, Singapore, smartphones

## Abstract

Widely and intensively used digital technologies have been an important feature of international responses to the COVID-19 pandemic. One especially interesting class of such technologies are dedicated contact and tracing apps collecting proximity data via the Bluetooth technology. In this article, I consider the development, deployment and imagined uses of apps in two countries: Singapore, a pioneer in the field, with its TraceTogether app, and Australia, a country that adapted Singapore’s app, devising its own COVIDSafe, as key to its national public health strategy early in the crisis. What is especially interesting about these cases is the privacy concerns the apps raised, and how these are dealt with in each country, also the ways in which each nation reimagines its immediate social future and health approach via such an app.

## Introduction

A striking feature of the COVID-19 pandemic has been the use of and appeal to digital technologies – fusing together what these technologies might offer in terms of efficacious communication and public health responses to help individuals and communities cope and contain the pandemic, on the one hand, as well as extending resources for social practices, expression, making sense, persisting with and reconfiguring rituals, and conjuring with the profound affective dimensions wrought by illness, death, loss, fear and isolation, on the other.

In the pandemic, digital technologies have been used across societies, in a way that harked back to earlier ideas from the 1990s of social life being nigh wholly dependent on life in ‘cyberspace’, and ‘virtual communities’. With widespread access to and ownership and use of Internet, mobile phones, social media, data, artificial intelligence (AI) and associated technologies already deeply, if very unequally, distributed globally, especially in middle- and high-income countries, the inception of the pandemic saw extended reliance on digital technologies – where terms of digital inclusion allowed for it. In a number of countries, governments also took the opportunity to issue calls to the acceleration of digitalisation, especially across groups and demographics where digital inclusion and take-up had been low, due to infrastructure, literacy and education, information and affordability.

One stand-out area in this regard was apps. Apps have been around since the 1980s and 1990s; however, it was the ‘smartphone moment’ of the launch of the Apple iPhone in 2008, and subsequent development of the apps for Apple mobile operating system devices (iOS) and launch of its Apps store, that kicked off the process by which apps became an integral part of everyday life for billions of users (Goggin, 2011; Miller and Matyivenko, 2014; Morris and Murray, 2018). From 2009 until the present day, technology companies around the world have offered their own apps and apps store, first with the ‘app store’ wars of 2011–2012 featuring many of the handset vendors that were household names in the worlds of 2G and 3G mobiles such as Nokia and Blackberry. Competition was much more suggestion in China, evidenced by the many Chinese app stores that dominate its huge market, and are significant distribution points for many users and communities internationally – especially given digital technologies being at the centre China’s external trade, finance and soft power ‘going out’ (Keane and Wu, 2018). Thus, apps are key to what has been recently called ‘infrastructural imaginaries’ (Nielson and Pedersen, 2015; see also Anand et al., 2018; Athique and Baulch, 2019; Mansell, 2012). So it is no surprise that apps formed a key part of the infrastructures woven into the pandemic, but also a specific, highly visible and ‘normalized’ response (Hoffman, 2020), in the form of dedicated apps – especially for tracing people and their ‘contacts’.

Apps were used for many significant purposes during the pandemic. Existing popular apps such as WhatsApp were used in some countries to send official government messages and distribute crucial public health information. The data sets generated by smartphones, computers, apps and people’s use of them, such as that data collected by Apple and Google, were used by public health officials, researchers and journalists to map population or district-level activity and movement, leading to the very interesting charts, graphs and visualisations in news and current affairs reports and features seeking to map and analyse the spread of COVID and its impact on social and economic activity. Apps allied with machine learning and AI were also used by medical researchers and clinicians to assist in the diagnosis of COVID, by asking millions of users to track and enter their symptoms, diary-like, to offer a way of pinpointing when someone might have become positive. Among the many varieties of COVID-dedicated apps were apps devoted to the purpose of tracking people and their potential contacts, in case they contracted the virus. So many countries developed apps for tracking and contact tracing, with so many prototypes in development and implemented, that MIT launched a Contact Tracing App Database (https://www.scl.org/news/11940-the-mit-contact-tracing-app-database), based on key questions from American Civil Liberties Union (ACLU) White paper (ACLU, 2020), to provide an authoritative reference point for those seeking to find their way through the claims and counter-claims of effectiveness. Apple and Google joined forces to amend their policies and create a joint protocol to make it easier for countries to use such data for contact tracing via apps (Michael and Abbas, 2020).

A full treatment of COVID contact tracing apps is outside the scope of this article (see, for instance, Cattuto et al., 2020; Hoffman, 2020; Vinuesa et al., 2020). Instead I focus on two especially interesting cases that offer us early insights into the socio-technical dynamics at play in such apps and the pandemic itself. These are Singapore’s TraceTogether app and Australia’s COVIDSafe app.

Asian countries were often referred to for their decisive and often authoritative responses to the pandemic. However, it was Singapore that attracted considerable early notice for its pioneering role in developing a particular kind of COVID contact tracing app – that captured the imagination of many other countries. Singapore was a pioneer in the development of COVID Bluetooth app in the form of its TraceTogether app. What was less publicised was that, shortly after launch of TraceTogether, Singapore changed tack. This modification of the app deployment and promotion, and place in the overall public health strategy, was less evident outside the city-state. Instead, Singapore’s TraceTogether app became a stand-out model for other countries, rather than the various other apps being implemented around the world such as those developed by the United States, South Korea, China, India or Israel (Babones, 2020).

Australia comes into the picture because it is Australia who first and most systematically sought to build on the TraceTogether model, including its privacy safeguards, with its own COVIDSafe app. In the capstone analysis of their series of timely interventions into the privacy debates on the introduction of Australia’s COVIDSafe, leading privacy scholars Graham Greenleaf and Katherine Kemp (2020) note, ‘Australia’s experiment is further advanced than most [countries] that are attempting to build a system based on voluntary uptake, protected by legislation (abstract, para 5). The Australian government sought to deploy COVIDSafe as a centrepiece of its effort to re-open Australian society after the national and state lockdowns occasioned by the ‘first wave’ of infections from March to May 2020. Where public concern regarding and discussion of privacy issues was clearly presented but publicly muted in Singapore, in Australia there was furious debate.

To explore the emergence, dynamics and implications of these two COVID apps, I will proceed as follows. First, I introduce and discuss Singapore’s TraceTogether, its development and first phase of take-up and deployment. Second, I turn to Australia’s COVIDSafe and consider its fast journey from incubation and policy idea to the touchstone to warrant the country’s re-opening, a veritable ‘national service’ (as Prime Minister Morrison couched it). Third, I return to Singapore, to discuss the rebooting of TraceTogether, after nearly 3 months of tepid take-up, as that country’s leadership sought to reassure its population that conditions were safe to re-open social life. Finally, I offer concluding remarks about COVID apps, social and technological imaginaries and digital media, as the nation state returns (Flew et al., 2016), and seek to gauge and exert its brittle powers, in a still deeply interconnected world.

## Singapore’s TraceTogether: ‘a fair degree of privacy’

To great fanfare, a dedicated contact tracing app was unfurled as a breakthrough in monitoring outbreaks of COVID-19 at the population level. While many teams around the world produced similar versions, the Singapore government rolled out the first such app – called ‘TraceTogether’. TraceTogether is an open source app based on Bluetooth, using the ‘Bluetrace’ protocol devised by a Singapore government team led by the GovTech agency – who have a track record of developing new kinds of open government apps, such as the Parking.SG app. In a 2018 interview, for instance, Janil Puthucheary (2018), Minister-in-charge of GovTech, discussed how the ‘GovTech guys, as a result of having to do the code for the service . . . are having to . . . hack policy’. Puthucheary explained, ‘You have to be able to codify the policy’, however that ‘some of our governmental processes and regulations result in extremely inelegant code’ (Puthucheary, 2018: 20′ 25″, 20′ 42″).

TraceTogether was made available for adoption elsewhere via GitHub. It is a combination of centralised contact tracing and follow-up (undertaken by government health authorities) and ‘decentralised contact logging’. The user downloads the app and activates Bluetooth on her device. The app can then detect another device in its vicinity, exchanging proximity information. To do so, the app uses information generated by the Bluetooth Relative Signal Strength Indicator (RSSI) readings that occur between devices over time to estimate proximity and duration of an encounter between users (Team TraceTogether, 2020c). If a person fell ill with COVID-19, they could grant the Ministry of Health access to gather their TraceTogether Bluetooth proximity data – to assist in contacting people who had close contact with the infected app user. For their part, the developers emphasised their view that TraceTogether would ‘complement contact tracing, and is not a substitute for professional judgement and human involvement in contact tracing’ (Team TraceTogether, 2020d). Interestingly, they also underscored that the ‘hybrid model’ of decentralised and centralised approach is what they feel ‘works for Singapore’ and that they ‘built it specifically for Singapore’ (Team TraceTogether, 2020b).

Released on 20 March 2020 by the Ministry of Health and GovTech (Baharudin and Wong, 2020), TraceTogether received over half a million downloads in its first 24 hours. A month later, the Singapore government claimed the app had achieved a 20% adoption rate – some 1.1 million users, of an overall estimated population of 5.7 million users (Team TraceTogether, 2020a). Upon launch in Singapore, there was relatively little public discussion of the privacy implications of TraceTogether in mainstream media and fora – although there was considerable disquiet, criticism and debate evident in blogs, social media and elsewhere.

For the most part, this is due to the structure and dynamics of Singaporean society, and its political arrangements, public policy traditions and strong systems of social control and clear support for or alternatively discouragement and sanctioning of different kinds of expression and voices – something well established in the scholarly literature (Chua, 2017; George, 2000, 2017; Lee, 2010, 2014), especially via various studies published in *Media International Australia* (most recently, Lee and Lee, 2019). In recent years, the Singapore government, following the dampened level of votes received by governing People’s Action Party (PAP), that has ruled since the 1967, in the 2011 Election, and a more sceptical populace (Barr, 2016; Zhang, 2016), it has sought to extend consultation and formal ‘listening’ mechanisms to provide additional opportunities for citizens’ voices. Furthermore, while there has been increased discussion of privacy with the rise of digital technologies and unprecedented expansion of data generation, collection and use, the legal and regulatory framework is relatively weak in relation to privacy rights taken-for-granted in many jurisdictions (Chesterman, 2012, 2018), even in the wake of the European General Data Protection Directive (GDPR). However, as we shall see, such debate did build over some months, as TraceTogether evolved. What is also important to note is that the Singaporean government clearly acknowledged the strength of attitudes and importance of privacy and data protection concerns, and sought to anticipate debates by building in some level of privacy protection.

The vision of TraceTogether is that proximity data gathering is ‘done in a peer-to-peer, decentralised fashion, to preserve privacy’, and that it relies upon a ‘trusted public health authority, committed to driving adoption’ (Team TraceTogether, 2020c). The developers and government emphasised that the privacy safeguards in the TraceTogether app are in effect an effort of the longstanding ideal of ‘privacy-by-design’ (Hustinx, 2010). The government emphasised that the information was stored on a user’s phone for 21 days, and then deleted – and user’s phone numbers are not exchanged, no geolocation data, personal identification data are not exchanged, so, as Minister Puthucheary noted, ‘the engineering has preserved the privacy of the users from each other’– calling the app ‘fairly elegant’, in the way it ‘preserves a fair degree of privacy’ (Ng, 2020; see also GovTech, 2020). Almost immediately the app did receive notice and discussion internationally, as one of a growing number of examples of COVID-19 contact tracings apps raising privacy concerns (Hu, 2020).

Meanwhile in Singapore, TraceTogether downloads flatlined. This occasioned international deliberation, such as an article in the *Wall Street Journal* entitled ‘Singapore built a coronavirus app, but it hasn’t worked so far’ (Lin and Chong, 2020). The stalled downloads of TraceTogether brought into view the conversations about privacy concerns, and whether this was a factor in user’s lack of motivation to download the app. Another reason advanced was that the app posed challenges for battery draining, due to the need to keep phones on. This was a view put by the CEO of Singapore’s investment or sovereign wealth company Temasek Holdings, Madame Ho Ching who has a reputation as a prolific commentator on public affairs by dint of her regular controversial Facebook posts (Ho is also the wife of Prime Minister Lee Hsien Loong) in a Facebook post of 9 May on the problems with TraceTogether (Ho, 2020a).

Whether by design or dawning acceptance, the government eased back its public communication and encouragement for citizens and other residents alike to download and use TraceTogether. Instead, it encouraged businesses, organisations, government offices and other entities to use a range of techniques to gather information about people’s movements – especially when they visited or spend significant time in public places. Check-in was principally done via scanning of a national identity card or employment or work permit ID card, or via an app called SafeEntry. Based on scanning of QR codes specific to each location, the SafeEntry app, and the policy it supported, was comprehensively promoted by government. This contrasted with TraceTogether, which was only lightly promoted by the Singaporean government, with the major campaign at the outset of its launch.

Presumably, on a small island – city-state, with strong civil service corps, existing ID systems (SingPass), and tightly managed immigrant and foreign worker ID and records, and digital government and technology capabilities, this evolving contact tracing system did not need to premised on an app such as TraceTogether, which presumably government was happy to allow to ‘fail fast’, given the bugs it faced.

## Australia’s COVIDSafe app: ‘helping to save someone’s life’

Despite the effectively prototypical status of TraceTogether, one of the first jurisdictions to adopt the technology was Australia. Prime Minister Scott Morrison referred to such an app as a key requirement in Australia’s ability make its transition out of lockdown (Prime Minister et al., 2020). In a radio interview with national talk show host Alan Jones, Morrison’s language is instructive, because it imagines technology, especially automated technology, as taking the vagaries and morally dubious qualities of human agency out of the picture: ‘[W]e need to get an automatic industrial level tracing of the coronavirus . . . Now, we’ve been working on this automatic process through an app that can ensure that we can know where the contacts were over that infection period and we can move very quickly to lock that down’ (Morrison, 2020a: para 6).

This kicked off a heated debate about privacy implications, leading a high-profile member of his own Coalition government, rural parliamentarian Barnaby Joyce to declare that he would not be downloading the app – countered by many other public figures who promised to do so. Despite the widespread criticism and concern, there was also significant support with some 2 million downloads in the first day of its release, topping the 5 million mark in early May (Koslowski, 2020), then 6.13 million by 1 June 2020 (Meixner, 2020). These figures raise various concerns, such as whether those who download the app used or continued to use it. Also what the rate of downloads were in different parts of the nation (Slonim, 2020). Let alone whether the COVIDSafe app was playing a role in helping to trace contacts and find positive cases of the virus (Preiss and Dexter, 2020).

For the Prime Minister, the COVIDSafe app was a rhetorical centrepiece of his policy initiative to vouchsafe a loosening of restrictions and begin to repair the economic damage the virus caused:

The Chief Medical Officer’s advice is we need the COVIDSafe app as part of the plan to save lives and save livelihoods. The more people who download this important public health app, the safer they and their family will be, the safer their community will be and the sooner we can safely lift restrictions and get back to business and do the things we love. (Prime Minister et al., 2020: para 4)

While he drew attention to the voluntary, consent-based nature of the app, Morrison also sought to exert maximal symbolic pressure by framing adoption in patriotic terms, likening it to national service in wartime (and also not ruling out making it mandatory) (Gredley, 2020):

I’ll be calling on Australians to do it as a matter of national service. In the same way people used to buy war bonds, back in the war times, you know, to come together to support the effort . . . If you download this app you’ll be helping save someone’s life. (Morrison, 2020b: sec 2:30)

On 1 May 2020, Morrison announced that Australia had earned an ‘early mark’, with restrictions being lifted in a week. In doing so, he spoke of the download numbers being a ‘critical element’ in deciding to what extent the easing would occur: ‘Mr Morrison said not installing the app was like going into the “blazing sun” without wearing sunscreen’ (Armstrong and Minear, 2020).

Various commentators and researchers expressed their views on how to promote downloading and take-up of the app. In the *Australian Financial Review*, a piece by technology editor Paul Smith, entitled ‘Think like a founder’, reported, ‘Entrepreneurs and health technology experts have urged the government to adopt all the tricks of the start-up trade to get more Australians downloading the COVIDSafe contact tracing app’ (Smith, 2020). The Australian Chief Scientist through his Rapid Research Information Forum commissioned a brief on *Motivators for use of the COVIDSafe app*, supported by the Australian Academy of Humanities, with Professor Genevieve Bell as lead author, and various leading media, communications and humanities researchers among the contributing authors (Bell et al., 2020 (Disclosure: I was a peer reviewer of this brief)). The brief suggested that ‘Illustrating that COVIDSafe works as intended may assist decision-making for those yet to download the app’ (Bell et al., 2020: 5). It also concluded that

The stories we will tell about Australian responses to, and uses of, COVIDSafe will matter too. The voices of trusted figures, community leaders, healthcare workers and citizens will likewise inform the adoption, and continued use of, COVIDSafe. (Bell et al., 2020: 7)

Many of these ‘stories’ clustered about the public perceptions and debate about the privacy, data and surveillances implications of the COVIDSafe app (Bell et al., 2020), driven by long-standing sensitivities and attitudes of Australians concerning privacy. Stretching back to the infamous and ill-fated Australia Card proposal of 1985, citizens’ privacy concerns had been more recently exacerbated by the Federal government’s poor handling of the ramp-up of its national e-health records registration system, MyHealth, which switched from an ‘opt-in’ to ‘opt-out’ basis in 2017 (Komesaroff and Kerridge, 2018; Goggin et al., 2019). With much at stake in terms of public health concerns at a critical juncture of the COVID pandemic, the Australian government emphasised that it was keen to adopt a ‘consent-based’ model, hence its interest in adapting the Singapore TraceTogether app. The government sought a formal Privacy Impact Statement from a leading law firm – which it published, with a detailed response from the Department of Health (2020b; Maddocks, 2020). This Privacy Impact documentation put important details of the workings of the COVIDSafe application, and the production, storage and handling, of resulting user data on the public record.

In response, the government emphasised that participation would be voluntary (Department of Health, 2020a); however the Privacy Impact Statement noted the potential for third-parties such as workplaces or businesses put pressure on or require people to use the app (Maddocks, 2020). Deleting the app would also delete the data stored on a user’s device, but not data in the national Data Store (however, the government guaranteed that all data held would be deleted at the end of the pandemic). The government was at pains to reassure the public on the secure hosting of the COVIDSafe Data Store, undertaken by Amazon Web Services (AWS). Their guarantees related to the data privacy and security obligations applying to AWS, but also to any prospect that such data might be requested and commandeered by the US government (given AWS is headquartered in the United States, and subject to their laws).

Over some weeks a furious debate ensued, and the Australian government proposed legislation to address the key concerns. This safeguard took the form of the Privacy Amendment (Public Health Contact Information) Act 2020. The bill quickly passed through the House of Representatives and the Senate and received assent on 15 May 2020. The Act creates several serious offences dealing with COVID app data, including ‘non-permitted, use, or disclosure’, ‘uploading COVID app data without consent’, ‘retaining or disclosing uploaded data outside Australia’, ‘decrypting encrypted COVID app data’ and ‘requiring participation in relation to COVIDSafe’ (Privacy Amendment, 2020: 4). ‘COVID app data’ is defined as ‘data relating to a person . . . collected or generated . . . through the operation of COVIDSafe’ and is either ‘registration data’ or ‘is stored, or has been stored . . . on a communication device’ (s. 95D (5) (a-b), Privacy Amendment, 2020: 8–9).

While the bill was passed containing significant safeguards, it contained serious flaws. As summarised by Greenleaf and Kemp, these included key information upon which the law was based and would operate was not made available to the public, including advices to the Minister upon which he relied to make the earlier Determination, and, crucially, the agreements between the Commonwealth and states and territories regarding the operation of the COVIDSafe app, and collection and sharing of app data; lack of public assessment of the law by the Federal and states and territories Privacy Commissioners; and only the source code for the COVIDSafe app was released, not the code for the National COVIDSafe Data Store (i.e. the server-side of the system, where security and privacy issues often manifest) (Greenleaf and Kemp, 2020). In addition, Greenleaf and Kemp critique the narrow focus of the *Privacy Act* amendment on ‘COVID app data’, suggesting instead that what is being created is an information system they dub the ‘COVIDSafe system’ (Greenleaf and Kemp, 2020).

As well as the specific defects of the new law, then, the major issue it raises is precisely the one feared by many experts and members of the public alike: that the app-based contact tracing represented by COVIDSafe, and other apps around the world, represent a deepening of technologies of surveillance in social life. While such apps and measures in which they are embedded are justified as exigent public health measures crucial in the emergency conditions of a pandemic, there is well-founded fears that this increase in surveillance will not be automatically or easily rolled back once countries feel the threat of COVID is ended or at least contained.

## Return of TraceTogether: Singapore’s ecology of COVID-19 apps, technologies and data

As Australian debates over COVIDSafe privacy subsided, there was a slow return to TraceTogether emerging in Singapore as the country’s leadership gingerly considered how to effect its re-opening from its 2-month circuit breaker. A task all the more urgent, given the ruling party’s dwindling time to call a national election.

Singapore’s was regarded an international model of wise and swift response with its handling of its ‘first wave’ of infections. However, in the second week of March 2020, Singapore tightened its measures, enacting a general shutdown and stay-at-home policy that it dubbed a ‘circuit breaker’. Initially the circuit breaker was announced to last for 1 month, but with the rising tide of positive cases in the crowded migrant worker dormitories, the government quickly extended for a second month.

A disturbing feature of Singapore’s data gathering and public reporting and communication during this period was the distinction clearly drawn and maintained in the daily bulletins between; cases in the migrant worker dormitories; and ‘community cases’ (these community cases were in turn divided between figures on Singapore citizens, permanent residents (PRs), migrant workers on work permits and workers on employment passes) (Han, 2020; Palma, 2020). The migrant workers were quarantined in the dormitories, with many then moved to across other repurposed facilities. And the numbers of cases were similarly quarantined, in a communicative-epistemological manner, to emphasise that the ‘real’ community spread remained low (usually below 10 cases in the ‘community’). Those numbered among the community included citizens and PRs initially, but subsequently, foreign pass holders who do not reside in dormitories but lived among the regular population, became part of these statistics once the dormitory cases started subsiding.

Ahead of the planned end to the circuit breaker on 2 June, the government made some mention of TraceTogether at various times in its public communications. However, its main focus remained racking movement and individuals’ location via check-in at the public places and business still open, such as convenience stores and shopping centres, or in taxis and ride-hailing services, especially via the SafeEntry app discussed above.

As the re-opening loomed, there was increasing discussion in government, and in parliament, on measures that would need to be implemented to contain and reduce the number of infections via contacts with migrant workers, especially once they were allowed to more regularly leave the dormitories, where they had been quarantined during the circuit-breaker period, and so circulate in the ‘community’. The government announced a new app, SGWorkPass, to ‘show which migrant workers can leave their dormitories for work’ (Ho, 2020). Workers will ‘get a “green status” on the app to indicate that their employer has been granted approval to resume operations, and that the dorm they stay in has been cleared’ (Ho, 2020). Otherwise, the app will show red to indicate they ‘cannot go out for work’ (Ho, 2020). This is reminiscent of the Chinese app, also adopted by India, which uses QRs, to show a user’s status as green (when they may enter offices, restaurants, malls or parks), or yellow (at risk) or red (strict quarantine) (Hu, 2020; India Today, 2020).

At this stage, TraceTogether returned – this time, as a central feature of the strategy. The government had been at pains to keep TraceTogether opt-in, with Foreign Minister Vivian Balakrishnan, also Minister-in-Charge of the Smart Nation Initiative, providing reassurance that the app would remain voluntary ‘as long as possible’ (Balakrishnan, 2020). In early June, Balakrishnan noted the problems with TraceTogether, including the technical issues with the app not running properly on Apple. As a result, he let it be known that Singaporean government was developing a ‘portable wearable device’ that will achieve the same end, that if it worked could be ‘distributed to everyone in Singapore’: ‘I believe this will be more inclusive, and it will ensure that all of us will be protected’ (Balakrishnan, 2020). The government emphasised that there would no ‘GPS chip’ on the device, nor any Internet connectivity. Even then, the TraceTogether token would need to be physically handed to the Health Ministry for uploading of the data, if a user tested positive for COVID-19 (Yu, 2020). The government’s keenness to be seen to address privacy was doubtless fuelled by a public backlash against the Token.

As policy researcher and commentator Carol Soon, from the Institute of Policy Studies, noted, ‘Within a short span of three days, a petition against the development of the device attracted about 30,000 signatories’ (Soon, 2020). Concerns of Singaporeans regarding data privacy were addressed in a report authored by her colleagues, which found attitudes vary according to the technology involved, illustrated by the finding that nearly 6 in 10 respondents supported use of CCTV to monitor people’s movements during the COVID ‘circuit breaker period’, but less than 50% were comfortable with having their mobile phone data tracked for contact tracing without their consent (Tay, 2020). To address such deep-seated concerns, Soon suggested the need for Singapore to urgently ‘achieve a working compromise between personal data and public good’, establishing principles and considering measures such as formation of a citizen’s panel for public deliberation (Soon, 2020). Regardless, the first batch of 10,000 TraceTogether tokens were distributed to seniors shortly on the eve of the 10 July 2020 general election – with officials from the Smart Nation and Digital Government Group (SNDGG) suggesting they were settling in for a long haul, saying the government will ‘continue to generate more awareness about the token among our prioritised population’ (SNDGG officials quoted in Yip, 2020).

## Discussion and conclusion

At the time of writing, the pandemic rages globally, and the career of COVID-19 contact tracing apps is still unfolding – with little evidence as yet of their efficacy. However, there are already clear grounds for concerns.

The strange thing about the Australian embrace of Bluetooth-based COVID tracing apps is how strongly it figured, for a time at least, as instrumental to the country’s public health response. Various commentators noted the irony that at the point COVIDSafe was being pushed upon the public, Australia was at a positive inflection point in terms of infections. As Greenleaf and Kemp note, this set the bar because other measures had already appeared to be successful in greatly abridging the spread of the virus (Greenleaf and Kemp, 2020: 6). The other obvious thing is that where apps did play a role in diminishing infection rates, these were not: (1) Bluetooth-based tracing apps, (2) and the apps used were integrated into a wider system of cross-referencing and marshalling personal identification and contact information and database systems (Greenleaf and Kemp, 2020). Yet the Australian government, for a short time at least, was very keen on the app as a symbolic game-changer in its public health approach to the pandemic – showing that it was taking charge. Rather like British Health Secretary, Matt Hancock some weeks later, when he promoted the English app-based test-and-trace system, telling the public ‘It is your civic duty’:

Do it for the people you love. Do it for the community. Do it for the NHS and do it for all the frontline workers . . . you’ll have the knowledge that when the call came you did your bit, at a time when it really mattered. (Hancock quoted in Bosley and Stewart, 2020)

In July 2020, there was an outbreak of COVID-19 cases that saw a lockdown re-imposed, and fuelled national concerns. At the time Victorian Chief Medical Officer Brett Sutton said the ‘app has not added a close contact’ that authorities had not already discovered via traditional contact tracing (Borys, 2020). Federal Health Minister Greg Hunt advised that at least 200 contacts nationally had been identified via the COVIDSafe app (Borys, 2020). For her part, NSW’s Chief Health Officer Kerry Chant, the state next in line for a potential resurgence of cases described the app as ‘one of the tools’, but not a ‘major feature’ in contact tracing (Borys, 2020). In his parsing of the app’s effectiveness, Australian Deputy Chief Medical Officer, Dr Nick Coatsworth, suggested that because of movement restriction, people had not been circulating, so the ‘app hasn’t identified those cases’, and that as ‘numbers go up then the app can come into its own’ (Coatsworth, 2020). With the groundswell for mask use in mind, Coatsworth (2020) cleverly sought to link the two, suggesting ‘if you are a supporter of mask use, you must also be based on the modelling, a supporter of downloading and activating the app’ (here he refers to the study by Sax Institute, see Currie et al., 2020).

For its part, Singapore took a less dramatic, more considered approach, especially in the first phase as it developed and launched its TraceTogether app. Singaporean leaders and health officials were also preoccupied with promoting the app to gain the maximum take-up and adherence. Yet, for reasons not entirely clear as yet, Singapore was reluctant to push the adoption of the app to the extent that Australia did – an interesting situation given earlier critiques of Singapore technocratic approach to health care, in particular (Barr, 2008).

As well as the privacy concerns that emerged in the second phase of the TraceTogether Token initiative, it may be that Singaporean actors thought the app was promising but not the main game. This would be because of the already well entrenched systems of requiring and using personal data, through an extensive infrastructure of technologies (including the CCTVs that featured in the IPS report), without the kind of concomitant privacy rights and practices that would be expected in some other jurisdictions such as Australia. The task of enlisting and normalising Singaporeans participation in these aspects of its surveillance-extensive ‘Smart Nation’ policies, over cumulative implementation of technology is taken to be essential, but it is increasingly fraught (Lee, 2020). In the first 6 months of pandemic response, then, the central element was Singapore’s established SingPass and other systems of identification cards and passes, which could be used in coordination with video recordings, and the wealth of digital data available from urban transportation systems, stored valued and transit cards, ride-hailing accounts and so on. As well as also as the citizen and netizen sousveillance and activism that saw recordings of potential miscreants breaching the regulations circulated online. In addition in the early weeks of the pandemic, identifying details of people’s residential locations, down to building numbers, were published in daily updates from Ministry of Health, and reprinted in media outlets.

Such measures point to the differences in privacy laws and protections in Singapore, as compared to Australia. Whereas Australian Privacy Act dates back to 1988, Singapore only enacted its first comprehensive law in 2012, the *Personal Data Protection Act*. At the time, legal scholar Simon Chesterman suggested that Singaporean had taken a ‘pragmatic approach’, potentially striking a balance between European and US approaches:

In Singapore, at least, reform is not being driven by the desire to defend the rights of data subjects; rather, it is based primarily on economic considerations, as well as the desire to position Singapore as a leader in the region for data storage and processing. (Chesterman, 2012: 414).

The Singapore laws and approach to privacy and data protection have not substantially changed since (Chesterman, 2018; Ong, 2020). Yet clearly citizens do have concerns – as the public response to the TraceTogether token suggest.

From a broader perspective, the return of TraceTogether to the fore of the Singaporean government’s strategy, especially to assist with the re-opening process after its ‘circuit breaker’, is very interesting indeed in the context of the country’s digitally underpinned governmentality (Ho, 2017; Lee, 2014, 2020; Willems and Graham, 2019). This is worth being in mind in interpreting the 2020 election, in which the government received some strong criticism by opposition candidates for its poor handling of the pandemic, especially concerning the continuing high number of cases in migrant worker dormitories. The PAP was returned to government, still with a ‘super majority’, of 83 seats out of the available 93. However, it was chastened by its share of the vote being reduced to 61.2% (from its 69.9% share in the 2019 election) – and an unprecedented 10 seats won by the opposition Workers’ Party (Loh, 2020). In the aftermath, the government has signalled its willingness on listening to electorate concerns, especially those of young voters (Yong, 2020).

All in all, in both these case studies, we see that the variations of the COVID-19 contact tracing apps, and the technical, social, policy and design dynamics of these, offer rich food for thought when it comes to understanding apps. Health information is an area of considerable sensitivity for most people. Trust is key, and with the widespread diffusion of mobile communication there has been considerable work on how to design and implement systems that can support cooperative and sustainable sharing of information between people and authorities to map the spread of infectious diseases (Lwin et al., 2014). However, it is now evident that the task of assembling appropriate social and cultural understandings of people’s lives and identities, their data selves (Lupton, 2020), the intricacies of technologies, the enmeshing of privacy expectations in design, and the construction of suitable legal, policy and governance arrangements, is challenging. In the COVID-19 pandemic, many countries across the world have had recourse to apps, as flexible agents with capacity to encode, materialise, represent and integrate such requirements, including some contradictory ones, and imagine and forge majoritarian supported social action. It is difficult not to see the turn to tracing apps as a pivotal moment in the expansion and entrenchment of surveillance technology in digital societies, of which Singapore in particular has been a leading example (Lee, 2020) – but is also playing out in contests and debates in many countries especially in Europe and Asia. How this ultimately turns out, and with what benefits for health, as well as legacies for democratic freedoms and daily life, we must wait and see.
